# Changes in physical fitness and body composition of athletes after the COVID-19 lockdown: a systematic review, meta-analysis, and meta-regression, with assessment of the certainty of evidence

**DOI:** 10.5114/biolsport.2026.153307

**Published:** 2025-10-01

**Authors:** Jad Adrian Washif, Khaled Trabelsi, Jeffrey Pagaduan, Marie Stella Louise Perreras, Imen Moussa-Chamari, Narimen Yousfi, David B. Pyne, Karim Chamari

**Affiliations:** 1 High Performance Branch, Sports Performance Division, National Sports Institute of Malaysia, Kuala Lumpur, Malaysia; 2 High Institute of Sport and Physical Education of Sfax, University of Sfax, Sfax, Tunisia; 3 Department of Movement Sciences and Sports Training, School of Sport Science, The University of Jordan, Amman, Jordan; 4 Melbourne School of Psychological Sciences, The University of Melbourne, Melbourne, Victoria, Australia; 5 Department of Sports Medicine and Science Graduate School, Konkuk University, Seoul, Republic of Korea; 6 Sport Coaching Department; College of Sport Sciences, Qatar University, Doha, Qatar; 7 Research Unit “Sport Sciences, Health and Movement”, Higher Institute of Sports and Physical Education of Kef, University of Jendouba, Kef, Tunisia; 8 Tunisian Research Laboratory “Sport Performance Optimisation”, National Center of Medicine and Science in Sport, Tunis, Tunisia; 9 Research Institute for Sport and Exercise, University of Canberra, Canberra, Australia; 10 Research Office, Naufar, Wellness and Recovery Center, Doha, Qatar; 11 Higher institute of Sport and Physical Education, ISSEP Ksar Saïd, Manouba University, Tunis, Tunisia

**Keywords:** Athletic performance, Body fat, Detraining, Modified exercise, Home training, Isolation, Resistance training, SARS CoV 2

## Abstract

This systematic review with meta-analysis analysed the effects of COVID-19 lockdowns on physical fitness and body composition in athletes. A comprehensive search was conducted in three databases (PubMed, Web of Science, and Scopus) up to January 2025 (included). Studies were included based on PICO criteria, involving adult athletes, original articles, and any quantitative assessment of physical fitness and/or body composition conducted within one month before and two weeks after the lockdown. The Joanna Briggs Institute Critical Appraisal Checklist was used to assess the risk of bias, while the Cochrane Grading of Recommendations Assessment, Development, and Evaluation (GRADE) approach evaluated the certainty of evidence. A total of 14 studies (261 athletes) with a low risk of bias met the inclusion criteria. Narrative synthesis revealed that the effects of lockdowns on athletes’ physical fitness and body composition were varied, with consistent impairments (e.g., endurance-related fitness), relative stability (e.g., body mass, CMJ height, maximal strength), and mixed results (e.g., sprinting). A meta-analysis of 11 studies indicated a non-significant effect of lockdown on body mass (effect size [ES]=−0.115, 95% confidence interval [CI] −0.214 to 0.164, P=0.797). Similarly, 10 studies showed a variable, non-significant reduction in CMJ height (ES=−0.303, 95% CI −0.655 to 0.045, P=0.097). However, CMJ relative peak power (six studies) demonstrated a trivial-small negative effect (ES=−0.199, 95% CI −0.341 to −0.058, P=0.019). These findings should be interpreted with caution as the certainty of evidence was very low. While evidence remains limited, targeted and individualised training might help mitigate some of the detraining effects observed during a lockdown, particularly in endurance-related fitness outcomes.

## INTRODUCTION

The coronavirus 2019 (COVID-19) pandemic triggered widespread restrictive actions in early 2020, leading to the closure of non-essential facilities including schools, restaurants, businesses and sports operations [1, 2]. These government-mandated public health measures (e.g., quarantine, curfew, or stay-at-home orders; termed simply “lockdown” from here on) substantially disrupted daily living, and general physical activity including athletes at all levels [1, 2, 3, 4, 5]. Athletes, including those at elite and professional levels, who depend on structured training environments and team settings, experienced substantial changes to their daily/regular routines [5, 6, 7] and (lacked) the usual medical care [8]. Many athletes engaged in home-based training, which was often deemed less effective for maintaining physical and mental well-being [2, 7, 9].

A substantial number of studies have documented the effects of a COVID-19 lockdown on training across a wide range of sports. During this period, athletes strove to maintain pre-lockdown training frequency, volume, intensity, and coaching-related aspects [10, 11, 12, 13], although data from 12,526 athletes highlighted a focus on maintaining or developing general fitness/health [2]. Regardless of this, athletes adopted improvised training methods that relied on limited equipment and bodyweight exercises [14, 15, 16]. These exercises were often carried out with inadequate stimulus to maintain physical fitness [2, 10], due to training monotony, reduced motivation, and sub-optimal conditions [17]. In most cases, athletes engaged in minimal or no specific training [11, 16], resulting in a substantial reduction in overall training volume e.g., 55% [11]. Adaptations varied in effectiveness, with challenges differing by athlete level, sport type and sex [18, 19, 20]. For instance, elite athletes had greater support and access to sporting facilities than their sub-elite counterparts [2, 21]. Athletes in weight-categorised and aquatic sports faced difficulties maintaining ideal body mass and training routines [22, 23]. Furthermore, female athletes experienced substantial reductions in training days and hours [24], and an increased risk of decline in their mental health outcomes (e.g. augmented anxiety) [25, 26].

Studies on the impact of lockdowns on physical fitness and body composition have yielded mixed findings [27, 28]. Declines in shoulder strength and range of motion were observed in handball players [29]. Similarly, poorer strength, power, sprinting, agility, aerobic fitness, and negative changes in body composition were reported among amateur football players [30]. In contrast, professional football players maintained fitness levels [27], as did highly-trained female football players, who preserved strength and agility through group-based interventions [31]. Among top-level handball players, explosive performance was retained but there was a reduced aerobic capacity [14]. Rampinini et al. [15] reported aerobic fitness improvements but no gains in anaerobic power, while Leo et al. [32] found no significant changes in power output or ${\dot{\text{V}}\text{O}}_{2\max}$ among elite cyclists. Interestingly, reduction of physical activity did not contribute to weight gain of professional soccer players [33]. These variations, likely due to methodological inconsistencies and/or differences in training pattern [16], highlight the need for a systematic review to synthesise findings and identify key trends.

Many studies on COVID-19 lockdown effects lacked consistent testing timelines, such as baselines often set days to months prior to lockdowns [10, 32, 34, 35, 36]. Some studies even compared postlockdown physical fitness and body composition with the post-transition period [37], which represent a distinct scope. A previous systematic review and meta-analysis did account for this issue and included studies with participants under 18, with less stringent criteria potentially affecting the generalisability of the findings [16].

Moreover, the meta-analysis of Rosa et al. [16] did not assess the certainty of evidence, which is an important methodological limitation under PRISMA 2020 (preferred reporting items for systematic reviews and meta-analyses) [38]. Importantly, with the lack of certainty of evidence assessment, the strength and reliability of findings remain uncertain, which can limit practical applications. The impact of non-supervised vs. supervised home training on athletes’ fitness retention post-lockdown remains uncertain as well [35]. Moreover, demographic (e.g., age, country) and contextual (e.g., sport type, athlete level, lockdown duration) variables may moderate the impact of lockdown on athletes. These variations can complicate cross-study comparisons. We addressed the gaps by systematically reviewing the effects of lockdowns (with or without interventions) on physical fitness in adult athletes, with a secondary focus on body composition, to better understand the impact of a lockdown on athletes and provide evidence-based guidance for managing future such disruptions in the future.

## MATERIALS AND METHODS

The study has been conducted in adherence to the PRISMA 2020 guidelines [38]. An a priori protocol was registered in the Open Science Framework database (Identifier: https://osf.io/37tue).

### Search strategy and eligibility

**FIG. 1:**
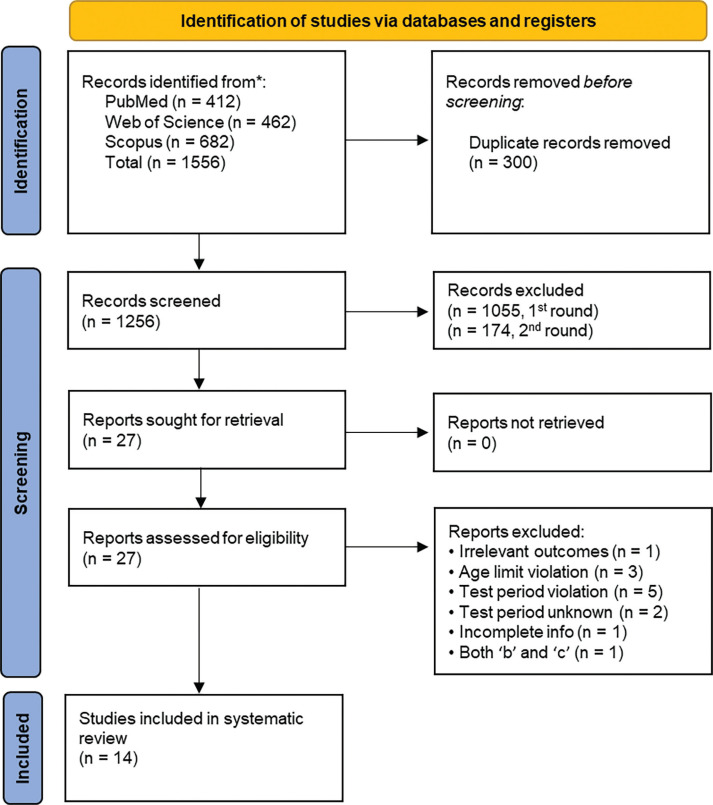
Flow chart of COVID-19 studies (main databases) for determining the effect of lockdown on physical performance measures and body composition

To facilitate the identification of relevant literature, an extensive search was conducted across three main databases including PubMed, Web of Science, and Scopus (Figure 1). The electronic database search was supplemented by a manual search, using backward (reference lists) and forward (cited articles) strategies to further identify relevant articles. A database containing grey literature, Google Scholar, was also searched (first 100 pages of results reviewed). We employed a publication timeframe spanning from February 2020 (onset of the COVID-19 pandemic) to January 2025 (5 years). In order to ascertain pertinent studies that have reported the effects of COVID-19 lockdown on physical fitness and body composition in athletes, specific keyword combinations were applied to the databases referenced (Table 1).

**TABLE 1:** Main databases and searches used to identify relevant articles; n = 1556.

1	**PubMed**	**412**
	*Fitness qualities and athletic performance*	
	((SARS-CoV-2 [Title/Abstract] OR COVID-19 [Title/Abstract] OR lockdown [Title/Abstract] OR confinement [Title/Abstract]) AND (Athlete* [Title/Abstract] OR player* [Title/Abstract])) AND (Performance [Title/Abstract] OR strength [Title/Abstract] OR power [Title/Abstract] OR endurance [Title/Abstract] OR sprint* [Title/Abstract] OR jump* [Title/Abstract] OR throw* [Title/Abstract] OR agility [Title/Abstract] OR flexibility [Title/Abstract])	356
	*Body composition*	
	((SARS-CoV-2 [Title/Abstract] OR COVID-19 [Title/Abstract] OR lockdown [Title/Abstract] OR confinement [Title/Abstract]) AND (Athlete* [Title/Abstract] OR player* [Title/Abstract])) AND (“Body composition” [Title/Abstract] OR bodyweight [Title/Abstract] OR “body mass” [Title/Abstract] OR “lean body mass” [Title/Abstract] OR “fat mass” [Title/Abstract])	56

2	**Web of Science**	**462**
	*Fitness qualities and athletic performance*	
	((AB=(SARS-CoV-2 OR COVID-19 OR Lockdown OR Confinement)) AND AB=(Athlete* OR Player*)) AND AB=(Performance OR Strength OR Power OR Endurance OR Sprint* OR Jump* OR Throw* OR Agility OR Flexibility)	400
	*Body composition*	
	((AB=(SARS-CoV-2 OR COVID-19 OR Lockdown OR Confinement)) AND AB=(Athlete* OR Player*)) AND AB=(“Body composition” OR Bodyweight OR “Body mass” OR “Fat mass”)	62

3	**Scopus**	**682**
	*Fitness qualities and athletic performance*	
	TITLE-ABS-KEY ((sars-cov-2 OR covid-19 OR lockdown OR confinement) AND (athlete* OR player*) AND (performance OR strength OR power OR endurance OR sprint* OR jump* OR throw* OR agility OR flexibility)) AND PUBYEAR > 2019 AND PUBYEAR < 2025 AND (LIMIT-TO (DOCTYPE, “ar”)) AND (LIMIT-TO (LANGUAGE, “English”))	573
	*Body composition*	
	TITLE-ABS-KEY ((sars-cov-2 OR covid-19 OR lockdown OR confinement) AND (athlete* OR player*) AND (“Body composition” OR bodyweight OR “Body mass” OR “Lean body mass” OR “Fat mass”)) AND (LIMIT-TO (DOCTYPE, “ar”)) AND (LIMIT-TO (LANGUAGE, “English”))	109

4	**Google Scholar**	**1000**
	“physical fitness” OR “athletic performance” OR “body composition” AND athletes AND “COVID-19 lockdown”	
	*Note: Google Scholar was considered as an additional database/source; however, it does not offer a built-in feature to download article titles for subsequent screening as in PubMed, Web of Science, and Scopus.*	

***Inclusion criteria.*** The PICO model for establishing eligibility criteria was utilised to present the criteria [39]: participant (P), intervention (I), comparison (C), and outcome (O). To be eligible for inclusion in the final analysis, a study had to meet the inclusion criteria. First, the article had to be a full-text, peer-reviewed, written in English, and published between 2020 and January 2025. Next, studies were included if they met the following criteria:

– P – adult athlete participants without a disability (≥ 18 years);
– I – original studies on athletes, including observational (e.g., prospective or retrospective cohort, longitudinal, cross-sectional) and experimental designs (e.g., randomised or non-randomised trials);
– C – reported changes (pre-post) during the early COVID-19 lockdown; pre-testing was conducted within *one month* before lockdown, and post-testing within *two weeks* post-lockdown;
– O – focused only on quantitative changes in physical fitness and selected performance metrics (i.e., strength, power, endurance, sprinting, jumping, throwing, agility/change of direction, and/or flexibility), as well as body composition (i.e., mass, lean body mass, fat mass, and/or skinfolds).

Studies were excluded if the following elements were present: (i) non-human and non-athletic population studies; (ii) non-observational study design such as qualitative study, editorials, opinion pieces, letter-to-editor, perspectives, commentaries, review, systematic review, and meta-analysis; and/or (iii) non-physical, non-objective measures (e.g., perception of changes in athletic performance and body composition).

### Data collection and selection process

Following the preliminary identification process, an assessment of each study was conducted. Mendeley Desktop software (V2.130.2, Elsevier, USA) was used to check for duplicates. A descriptive methodology was employed to assess the suitability of studies including all titles, abstracts, and full-text articles. Furthermore, two authors conducted an independent search and evaluation of studies using the reference lists of previously evaluated studies. Subsequently, based on the results of a cross-examination of the identified literature by each reviewer, studies were either included or excluded for further analysis. In instances of disagreement between the two reviewers, a third independent reviewer was consulted for resolution.

### Risk of bias assessment

This study employed a methodological assessment based on an evaluation quality scale to limit the possibility of bias in the studies selected depending on the study design [40]. The quality of the published papers was independently assessed using the JBI (Joanna Briggs Institute) Critical Appraisal Checklist for Quasi-Experimental Studies [41]. The JBI checklist consists of nine key questions (Table 2, Table S1). For each question, studies were rated as “yes” if they met the specific criteria, “no” if they did not, or “unclear” if the information provided was insufficient to make a definitive judgment. The following thresholds were established based on literature [42] and discussion: low risk of bias (‘yes’ scores ≥ 70%), moderate (50 ≤ ‘yes’ scores < 70%), and high (‘yes’ scores < 50%).

**TABLE 2:** Quality assessment of the included studies for body mass, CMJ height, and CMJ relative peak power using the JBI Critical Appraisal Checklist.

Body mass	1	2	3	4	5	6	7	8	9	RoB (%)
Ambrozy et al. [59]	Y	Y	Y	NA	N	Y	Y	U	Y	75
Anderson et al. [27]	Y	Y	Y	NA	N	Y	Y	Y	Y	88
Campa et al. [61]	Y	Y	Y	NA	N	Y	Y	Y	Y	88
Cohen et al. [55]	Y	Y	Y	NA	N	Y	Y	U	U	63
Fatih et al. [30]	U	Y	U	NA	N	Y	Y	U	Y	50
Sannicandro & Bisciotti [28]	Y	Y	U	NA	N	Y	U	U	Y	50
Scoz et al. A [60]	Y	Y	Y	NA	N	Y	U	Y	Y	75
Scoz et al. B [60]	Y	Y	Y	NA	N	Y	U	Y	Y	75
Spyrou et al. [56]	Y	Y	Y	NA	N	Y	Y	Y	Y	88
Tan et al. [54]	Y	Y	Y	NA	N	Y	Y	Y	Y	88
Villaseca-Vicuña et al. [57]	Y	Y	N	NA	N	Y	Y	Y	Y	75
Global risk of bias score (%)										74

*CMJ height*										
Anderson et al. [27]	Y	Y	Y	NA	N	Y	Y	Y	Y	88
Cohen et al. [55]	Y	Y	Y	NA	N	Y	Y	Y	U	75
Fatih et al. [30]	U	Y	U	NA	N	Y	U	Y	Y	50
Pedersen et al. [31]	Y	Y	U	NA	N	Y	Y	Y	Y	75
Rampinini et al. [15]	Y	Y	Y	NA	N	Y	Y	Y	Y	88
Sannicandro & Bisciotti [28]	Y	Y	U	NA	N	Y	U	U	Y	50
Spyrou et al. [56]	Y	Y	Y	NA	N	Y	N	Y	Y	75
Tan et al. [54]	Y	Y	Y	NA	N	Y	Y	Y	Y	88
Villaseca-Vicuña [57]	Y	Y	N	NA	N	Y	Y	Y	Y	75
Valenzuela et al. [58]	Y	Y	N	NA	N	Y	Y	Y	NA	63
Global risk of bias score (%)										73

*CMJ relative peak power*										
Cohen et al. [55]	Y	Y	Y	NA	N	Y	Y	Y	U	75
Fatih et al. [30]	U	Y	U	NA	N	Y	U	Y	Y	50
Pedersen et al. [31]	Y	Y	U	NA	N	Y	Y	Y	Y	75
Rampinini et al. [15]	Y	Y	Y	NA	N	Y	Y	Y	Y	88
Spyrou et al. [56]	Y	Y	Y	NA	N	Y	N	Y	Y	75
Tan et al. [54]	Y	Y	Y	NA	N	Y	Y	Y	Y	88
Global risk of bias score (%)										75

Note; Y: Yes; N: No; U: Unclear; NA: Not Applicable; RoB: Risk of Bias; Item 1: Is it clear in the study what is the ‘cause’ and what is the ‘effect’ (i.e. there is no confusion about which variable comes first)?; Item 2: Were the participants included in any comparisons similar?; Item 3: Were the participants included in any comparisons receiving similar treatment/care, other than the exposure or intervention of interest?; Item 4: Was there a control group?; Item 5: Were there multiple measurements of the outcome both pre and post the intervention/exposure?; Item 6: Was follow up complete and if not, were differences between groups in terms of their follow up adequately described and analysed?; Item 7: Were the outcomes of participants included in any comparisons measured in the same way?; Item 8: Were outcomes measured in a reliable way?; Item 9: Was appropriate statistical analysis used?;

### Certainty of evidence assessment

To rate the certainty of the evidence provided by this review, the Cochrane Grading of Recommendations Assessment, Development and Evaluation (GRADE) approach was employed [43]. This evaluation focused on three outcomes detailed in the meta-analyses (i.e., body mass, countermovement jump (CMJ) height, CMJ relative peak power). GRADE assessment involved examining the methodological quality of the studies (i.e., risk of bias), the heterogeneity of results across studies (i.e., inconsistency), the generalisability of the findings to the target population (i.e., indirectness), imprecision of estimates, and the risk of publication bias [43].

### Data extraction

The relevant information was retrieved after cross-examination to ensure its alignment with the study’s objectives. The following data were collected for each study: a. reference number; b. study characteristics (e.g., sample size, age [mean and/or range], country, athletic population (sport or sports), and assessment period); c. activities of home training or social isolation (structured or nonstructured); d. main results; and e. main findings (conclusion). For studies for which the required data were not provided in tables or texts, data were estimated from graphs using an online tool “web plot digitizer” (https://automeris.io/WebPlotDigitizer/) [44].

### Meta-analysis

The meta-analysis was conducted using Comprehensive Meta-Analysis (CMA) software (Comprehensive Meta-Analysis version 3.0, Biostat). A series of random one-group (pre/post) meta-analyses were performed to compare variables before and after the COVID-19 lockdown, utilising sample size, means, standard deviations, and pre/ post correlations. It is important to note that pre/post correlations were not available in all manuscripts. Therefore, following Rosenthal’s recommendations, we adopted a conservative estimate of r = 0.7 [45].

Effect sizes (ESs) with 95% confidence intervals (CIs) were calculated based on Cohen’s method, representing standardised mean differences (SMD) [46].

Forest plots were employed to illustrate points of the ES and 95% CIs. A negative ES value indicated a decrease in the COVID-19 lockdown outcome measure, while a positive ES value indicated an increased COVID-19 lockdown outcome measure. ESs were interpreted as trivial (ES < 0.2), small (ES 0.2– < 0.6), moderate (ES 0.6– < 1.2), large (ES 1.2– < 2.0), very large (ES 2.0– < 4.0), or extremely large (ES ≥ 4.0) [46].

Q [47] and *I*^2^ [48] statistics were used to assess statistical heterogeneity. An *I*^2^ value > 50% was regarded as evidence of substantial heterogeneity [48].

The Cochrane Handbook recommends conducting meta-regression only when there are at least ten studies in a meta-analysis [49]. For subgroup analyses, it is suggested that each subgroup should contain a minimum of four studies [50].

In accordance with these guidelines, moderator analyses were conducted to explore potential sources of variance and heterogeneity. For body mass, meta-regression was performed for continuous variables (i.e., lockdown duration, sample size, sample mean age, journal impact factor, risk of bias) and categorical variables (i.e., country), and subgroup analyses were conducted for categorical variables (i.e., dietary guidance, lockdown duration > 12 weeks, athletes status). Similarly, for CMJ height, moderator analyses were performed using meta-regression for continuous variables (i.e., lockdown duration, sample size, sample mean age, pre-lockdown CMJ performance, journal impact factor, risk of bias) and categorical variables (i.e., country), and subgroup analyses were conducted for categorical variables (i.e., dietary guidance, lockdown duration > 12 weeks).

Funnel plots’ potential asymmetries, Begg and Mazumdar’s rank correlation test [51], Egger’s linear regression test [52], and Duval and Tweedie’s trim-and-fill test [53] were conducted to evaluate publication bias. Leave-one-out sensitivity analyses were conducted to evaluate the stability of the pooled ESs by systematically excluding each study and assessing its influence on the overall findings. An a priori significance level of *P* < 0.05 was applied to all analyses.

## RESULTS

### Study selection

A search of electronic databases and a review of study reference lists revealed a total of 1556 studies (Figure 1). After assessing the duplicates, 300 studies were removed. We then screened the remaining studies against the inclusion requirements, and identified 201 potentially eligible papers. We further discarded 174 articles after extensive checks, with reference to both the inclusion and exclusion criteria. Subsequently, 27 full-text studies were deemed relevant, and full review of each paper was conducted. Further, some articles were deemed either irrelevant (n = 1); violating the test period (n = 5) or age threshold (n = 3) limits, or both (n = 1), and other reasons described in Figure 1. Articles were considered even if only partial details were available (e.g., results presented in figure format), except if they violated the inclusion criteria. We made an effort to obtain necessary information from the corresponding authors with limited success (eight authors were contacted, and only two responded). Finally, we evaluated 14 articles in our review (Table S1). The study selection process is detailed in Figure 1.

### Study characteristics

The participants’ characteristics of the 14 studies included in this systematic review were reported in Table S1. Briefly, (a) sample size: the 14 studies (involving 11 countries) comprised a total of 281 individuals (222 male and 59 female), with sample size ranging from 7 to 29; (b) participants were adult athletes aged (on average) between 18 to 31 years; (c) sports: 10 studies involved football players, and one study each on kickboxing, futsal, wushu, and badminton.

### Risk of bias

Of all studies (items) included in this systematic review, almost all showed low risk of bias (Table 2, Table S1). Six out of 27 items included in meta-analysis showed moderate risk of bias. One of the main concerns was whether the participants of a study received similar exposure (treatment/care) aside from the intervention of interest. The average score of all studies was 81%, which indicates an overall rating of low risk of bias.

### Outcomes of systematic-review

Among the 14 articles in this study (total athletes: 261), 10 investigated changes in body mass, four in body composition, and three in lean body mass. For performance or fitness measures, 10 examined changes in CMJ height, six compared changes in CMJ peak power, and three examined changes in 10 m sprint, maximum squat strength, and ${\dot{\text{V}}\text{O}}_{2\max}$. Metrics with at least three related variables were systematically reviewed to explore the patterns of change with COVID-19 lockdown.

- *Effects on jumping-related parameters.* Ten studies investigated changes in CMJ height, with three studies reporting a decrease [28, 30, 54], and seven studies found no change [15, 27, 31, 55, 56, 57, 58]. Furthermore, three studies reported changes in standing long jump, with one study reporting a decrease [59], and two studies finding no change [30, 56].
- *Effects on linear sprint performance.* Five studies investigated 10 m and/or 30 m and/or 50 m sprints. For 10 m sprints, two studies found poorer sprint performance [28, 56], and two studies reported no change [31, 57]. For 30 m sprints, one study found poorer performance [30], and one study no change [57]. For 50 m sprints, one study found no change [59].
- *Effects on maximum strength.* Three studies investigated back squat strength. For absolute back squat strength, one study reported a ‘decrease’ based on ES [58], and two studies found no change [31, 57]. For relative back squat strength, two studies reported no change [31, 57]. For handgrip strength, one study found a decrease [30], and another study reported no change [59]. In terms of isokinetic measures, one study reported a decrease in internal/external rotation of elbows [29], while another study reported no change in all but one of the assessed measures [60]. One study on eccentric hamstring strength also reported no change.
- *Effects on muscular endurance.* Two studies investigated muscular strength endurance, and both reported a decrease in maximal pull-up repetitions [59], and 30 s push-up repetitions [30]. One study reported no change in a core endurance test [59].
- *Effects on aerobic-related measures.* Three studies reported a reduction in ${\dot{\text{V}}\text{O}}_{2\max}$ [28, 30, 59]. Furthermore, one study reported improvement in post-lockdown with lower blood lactate levels in a Mognoni test [15], one study revealed maintenance of percentage of maximal heart rate reached in submaximal 30–15 IFT [27], and one study reported a decrease in Yo-Yo test performance [57].
- *Effects on flexibility.* Two studies reported a reduction in flexibility (forward bend / sit and reach) [30, 59], while one study on shoulder range of motion reported no change in most variables except dominant shoulder internal rotation [29].
- *Effects on body mass and composition variables.* Ten studies investigated changes in body mass, with three studies demonstrating an increase [30, 57, 59], two studies a decrease [28, 61], and five studies no change [27, 54, 55, 56, 60]. Three studies reported stability in muscle mass or lean body mass [54, 56, 57]. Four studies reported changes in fat mass or fat percentage, with one observing an increase [54], and three reporting no change [56, 57, 61]. Two studies reported no changes in skinfold thickness [27, 57].

### Outcomes of the meta-analysis

Only studies with adequate information were included in the metaanalysis. Information were either sourced from the original papers or relevant authors. A meta-analysis of metrics from at least 6 studies was conducted to investigate precise changes in variable measures.

**FIG. 2:**
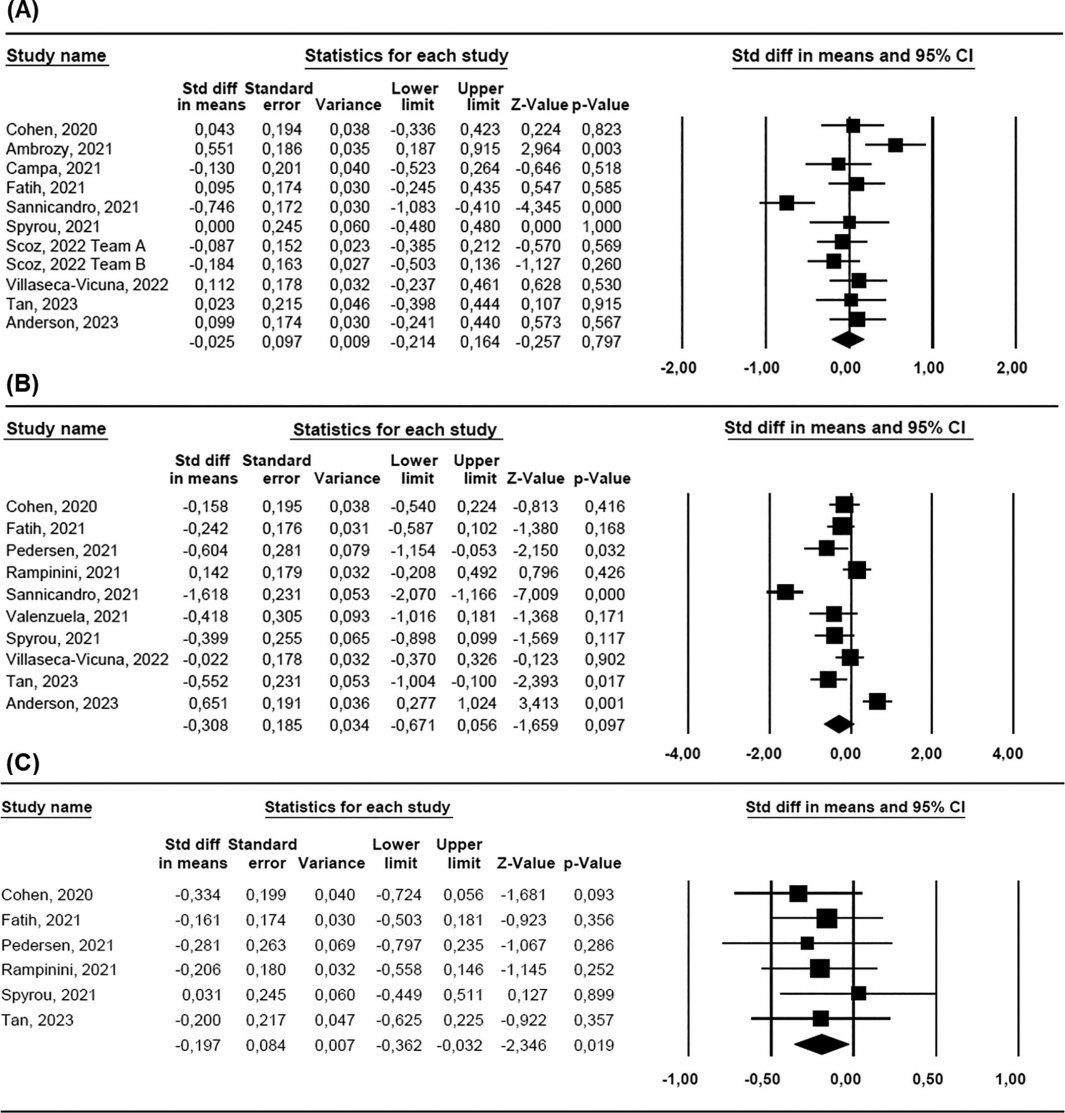
Forest plot for the changes in body mass (A), CMJ height (B), and CMJ relative peak power (C) after the COVID-19 lockdown

**FIG. 3:**
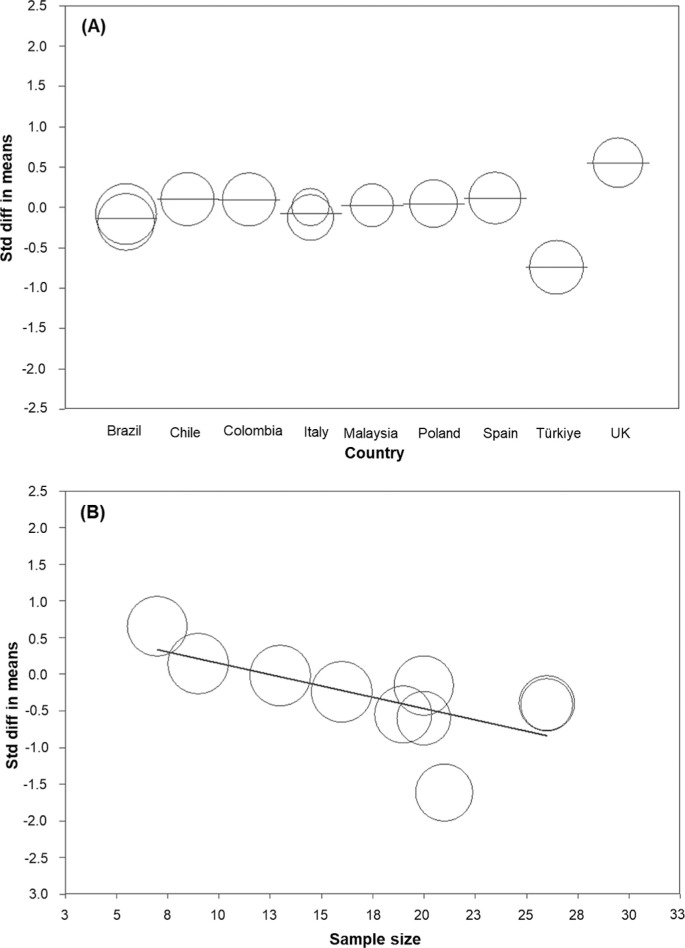
Meta-regression of body mass changes (SMD) by country (A), and CMJ height changes (SMD) by sample size (B)

**FIG. 4:**
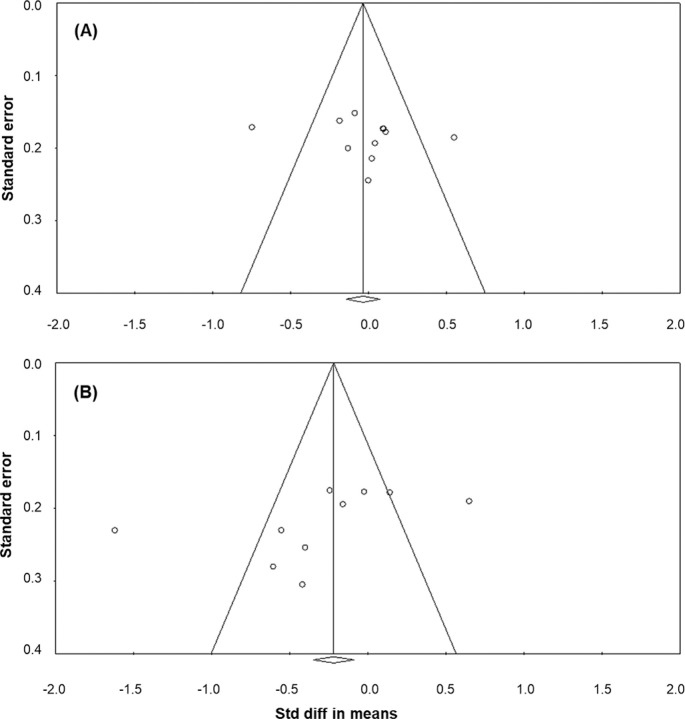
Funnel plot of standard error by standard difference in means for body mass (A) and CMJ height (B)

- *Body mass.* Ten studies [27, 28, 30, 54, 55, 56, 57, 59, 60, 61], involving a total of 208 participants, were used to characterise the change in athletes’ body mass with the lockdown. A meta-analysis of 11 ESs indicated a non-significant effect of lockdown on body mass (ES = −0.025: trivial, standard error [SE] = 0.097, and 95% CI −0.214 to 0.164, Z = −0.257, *P* = 0.797; Figure 2A), with a substantial statistical heterogeneity (Q = 30.356, df = 10, *P* = 0.001; *I^2^* = 67.1%). Meta-regression analysis stratified by body mass – country of study was statistically significant, indicating that athletes from Türkiye experienced the most pronounced changes, while those from the UK were the least affected (Q = 30.00, df = 8, *P* <0.001) (Figure 3). However, meta-regression based on lockdown duration (coefficient = 0.002, SE = 0.003, 95% CI −0.004 to 0.007, Z = 0.60, *P* = 0.552), mean sample age (coefficient = 0.006, SE = 0.03, 95% CI −0.005 to 0.114, Z = 1.81, *P* = 0.070), journal impact factor (coefficient = 0.037, SE = 0.056, 95% CI −0.073 to 0.147, Z = 0.66, *P* = 0.509), sample size (coefficient = −0.002, SE = 0.024, 95% CI −0.049 to 0.045, Z = −0.08, *P* = 0.934), and risk of bias (coefficient = 0.010, SE = 0.001, 95% CI −0.001 to 0.022, Z = 1.72, *P* = 0.086) did not yield statistically significant results.
  Subgroup analysis indicated that lockdown duration, whether greater than 12 weeks (ES = 0.135, SE = 0.113, 95% CI −0.409 to 0.104, Z = −1.165, *P* = 0.244) or less than 12 weeks (ES = −0.152, SE = 0.113, 95% CI −0.086 to 0.355, Z = 1.196, *P* = 0.232), did not significantly impact the outcome (*P* = 0.096). Similarly, subgroup analysis based on athlete status showed that the athletes’ level, i.e. whether neither amateur athletes (ES = −0.148, SE = 0.216, 95% CI −0.573 to 0.276, Z = −0.686, *P* = 0.493) nor professional athletes (ES = 0.051, SE = 0.106, 95% CI −0.155 to 0.258, Z = 0.488, *P* = 0.626) significantly influenced the outcome (*P* = 0.707).
  The funnel plot indicates evidence of publication bias (Figure 4A). However, Begg and Mazumdar’s rank correlation test (Kendall’s S: P-Q = 7.00; tau without continuity correction = 0.127, Z = 0.545, *P* = 0.293; tau with continuity correction = 0.109, Z = 0.467, *P* = 0.320) and Egger’s linear regression test (intercept = 2.788, SE = 4.457, 95% CI −7.294 to 12.870, t = 0.625, df = 9, *P* = 0.274) did not confirm this bias. The Duval and Tweedie trim-andfill test identified four missing studies, adjusting the ES to −0.166 (95% CI −0.254 to 0.022, Q = 58.415). Leave-one-out sensitivity analysis confirmed the robustness of the results, as the overall findings remained consistent regardless of which study was excluded (Figure S1).
- *CMJ height.* Ten studies [15, 27, 28, 30, 31, 54, 55, 56, 57, 58] involving 159 participants assessed athletes’ jump height in the CMJ before and after the lockdown. A meta-analysis of 10 ES statistics showed a non-significant variable effect of lockdown on CMJ height (ES = −0.308: small, SE = 0.185, 95% CI −0.671 to 0.056, Z = −1.659, *P* = 0.097; Figure 2B). Statistical heterogeneity was high (Q = 67.864, df = 9, *P* < 0.001; *I^2^* = 86.7%).
  Meta-regression stratified by the CMJ height-sample size was statistically significant (coefficient = −0.062, SE = 0.023, 95% CI −0.106 to −0.018, Z = −2.74, *P* = 0.006), indicating that larger sample sizes are associated with smaller ESs, while smaller studies report greater effects. However, meta-regression based on lockdown duration (coefficient = 0.002, SE = 0.003, 95% CI −0.004 to 0.007, Z = 0.60, *P* = 0.553), study country (Q = 3.09, df = 7; *P* = 0.877), and pre-lockdown performance (coefficient = −0.043, SE = 0.025, 95% CI −0.091 to 0.006, Z = −1.73, *P* = 0.084) did not yield statistically significant results. Subgroup analysis indicated that lockdown duration, whether greater than 12 weeks (ES = −0.506, SE = 0.264, 95% CI −1.024 to 0.012, Z = −1.914, *P* = 0.056) or less than 12 weeks (ES = −0.093, SE = 0.240, 95% CI −0.563 to 0.377, Z = −0.389, *P* = 0.697), did not significantly impact the outcome (*P* = 0.248).
  The funnel plot indicates potential publication bias (Figure 4B). However, Begg and Mazumdar’s rank correlation test (Kendall’s S: P-Q = −15.00; tau without continuity correction = −0.333, Z = 1.341, *P* = 0.090; tau with continuity correction = −0.311, Z = 1.252, *P* = 0.105) and Egger’s linear regression test (intercept = −7.304, SE = 4.418, 95% CI −17.492 to 2.884, t = 1.653, df = 8, *P* = 0.068) did not indicate potential publication bias. The Duval and Tweedie trim-and-fill test did not identify any missing studies. The leave-one-out sensitivity analysis demonstrated the stability of the findings, showing that the overall results were unaffected by the exclusion of any single study (Figure S1).
- *CMJ relative peak power.* Six studies [15, 30, 31, 54, 55, 56], involving 87 participants, were used to assess CMJ relative peak power before and after the lockdown. A meta-analysis of six ES statistics revealed a significant negative effect of lockdown on CMJ relative peak power (ES = −0.197: trivial-small, SE = 0.084, 95% CI −0.362 to -0.032, Z = −2.346, *P* = 0.019; Figure 2C), with a negligible statistical heterogeneity (Q = 1.491, df = 5, *P* = 0.914; *I^2^* = 0%). The findings remained consistent throughout the leave-one-out sensitivity analysis, indicating that no individual study significantly affected the overall results (Figure S1).

### Certainty of evidence assessment

The summary of the certainty of evidence using the GRADE approach is presented in Table S2 and indicates that the certainty of evidence for body mass, CMJ height, and CMJ relative peak power is at a critical level. Briefly, due to the observational design of these studies, the GRADE assessment started with a low certainty of evidence. The final GRADE results indicate very low certainty of evidence for all the outcomes, resulted from the downgrades in body mass (twice; small total number of participants and wide CI), CMJ height (twice; most studies’ ESs indicated no effect and small total number of participants), and CMJ relative peak power from imprecision related to the small total number of participants and wide CI.

## DISCUSSION

The outcomes of our systematic review and meta-analysis demonstrate that the impact of the COVID-19 lockdown on athletes varied across different physical fitness and body composition measures. First, we minimised the temporal bias by controlling assessment timelines, limiting the studies evaluated to; (i) a pre-lockdown assessment within one month prior to and (ii) post-lockdown assessments within two weeks of reopening. Meta-analysis findings indicate that body mass (ES = −0.115) and CMJ height (ES = −0.308) exhibited non-significant changes, whereas CMJ relative peak power showed a significant decline after lockdown (ES = −0.197). Furthermore, a review of the included studies revealed a mixed response in various physical fitness and body composition parameters, such as consistent decrements in muscular endurance, aerobic fitness, and flexibility, while muscular strength, sprinting, and jumping performance exhibited variable responses across studies. Similarly, the outcomes in body composition after lockdown were variable, with studies variously reporting an increase, decrease, or no change in body mass, muscle mass, and/or fat mass. Overall, these outcomes highlight the variability in physical fitness and body composition responses to the studied’ lockdowns.

### Effects on physical fitness

Our meta-analysis revealed no significant decline in CMJ height (ES = −0.308), aligning with previous findings and evidence that this variable remains unaffected by COVID-19 lockdown-related detraining [16]. The ability to preserve CMJ height (only *small* negative change observed, albeit with substantial variability) is likely attributed to the extent of home training and study’ methods characteristics [31, 15]. Our meta-regression also indicates no influence of pre-lockdown performance (nor lockdown duration) on the observed findings. Home-based activities typically included bodyweight-based jumping (in both vertical and horizontal planes) and muscular exercises aiming to maintain or enhance muscular and cardiorespiratory fitness [2, 9]. Not surprisingly, horizontal jump (for distance) was generally stable [30, 56], although one study reported a decrease in performance [59]. Importantly, variations were evident in initial strength levels depending on training background and sport. Athletes with lower baseline strength and power (e.g., CMJ height of ~35 cm for males [56] and ~29 cm for females [31] typically experienced negligible changes post-lockdown [16]. These findings in CMJ height should be interpreted in light of our meta-regression results, which identified an influence of sample size, where studies with larger sample sizes tended to report smaller ESs, and vice versa. Nevertheless, CMJ relative peak power (i.e., adjusted for body mass changes) may be more sensitive to negative effects of lockdowns as shown in our meta-analysis (ES = −0.197). This observation may indicate that while most athletes may retain jump height or distance, their power-to-weight ratio may have been “slightly” compromised, given the *trivial-small* decline found in CMJ relative peak power.

Beyond jump performance, declines were consistently observed across strength-related measures, sprint performance, and endurance-related capacity. Research shows that strength declines within three weeks of training cessation, with greater losses reported after five weeks, as observed in elite rugby and American football players [62]. In cohorts with modest strength levels, no changes were observed in maximum strength variables [31, 57]. Similarly, professional soccer players showed no impact on most isokinetic strength variables, except for reduced eccentric knee flexor peak torque [60]. Poorer sprint time was evident even over a 10 m distance, e.g., from 1.91 to 2.09 seconds, a 9% impairment [28], which is indicative of reduced acceleration ability. This observation may be due to a decline in neuromuscular performance resulting from a lack of specific training (including volume and intensity) e.g., resisted sprint training (sled pull) as a means to enhance acceleration during home training [63]. Additionally, hamstring and back flexibility also decreased post-lockdown in two studies [30, 59]. Notably, reduced hamstring flexibility has been reported as a risk factor for acute hamstring injuries in soccer players [64].

As endurance capacity is crucial in many team sports, maintaining it through bodyweight exercises during lockdown seems an important practical tip [35]. Reduced muscular strength endurance (repetition performed to momentary failure) was reported in two studies due to lockdown, and concurrently, aerobic capacity/fitness (${\dot{\text{V}}\text{O}}_{2\max}$) also exhibited a similar reduction [30, 59]. Similarly, a study that tested young male soccer players’ 38 days prior to lockdown also found a similar outcome for aerobic capacity [65]. Reduced training stimuli leads to impairments in physiological determinants of endurance performance, including oxygen uptake, ventilatory efficiency, and cardiac output [66, 67]. Short-term (≤ 4 weeks) and long-term (> 4 weeks) detraining reduced ${\dot{\text{V}}\text{O}}_{2\max}$ by 4–14% and 6–20%, respectively, depending on initial fitness levels [67, 68, 69]. Nevertheless, difference regarding ${\dot{\text{V}}\text{O}}_{2\max}$ changes between 30–90 days detraining and more than 90 days was not significant [70]. Maintaining aerobic adaptations requires high-intensity sessions at 80–90% max heart rate or > 80% ${\dot{\text{V}}\text{O}}_{2\max}$ [67, 70, 71]. While endurance athletes may sustain some training adaptations through home-based modalities such as a treadmill, cycle roller, or rowing ergometer, these alternatives also depend on the access to equipment and the individual environment and living circumstances of athletes [2, 72].

Athletes adapt home-based training to counteract restrictive policies, often modifying exercises and using household items, which can affect fitness positively or negatively [2, 9, 72]. In a mini review, typical home training for team-sport athletes consisted of ~5 weekly sessions (45–90 min each), with a primary focus on muscular strength and endurance [9]. However, such practices did not adequately preserve ${\dot{\text{V}}\text{O}}_{2\max}$ and sprint times, while producing inconsistent (negative/positive) changes in CMJ height [9]. Given that HIIT (alternating intense exercise with rest) enhances aerobic adaptations while resistance training improves strength and explosive performance, these training modalities maybe practical during lockdown [27], particularly for sports requiring both explosive power and aerobic endurance. Studies showed that HIIT as part of home training [15, 27] generally appeared to preserve endurance-related parameters, along with several other variables. Moreover, based on Table S1, many studies without HIIT (or information not being reported in the manuscript) during lockdown still reported maintenance in different fitness qualities. Training specificity may be required to mitigate detraining effects [29, 63]. Moreover, some physical fitness might be retained if training cessation is short (≤ 4 weeks) [67, 73, 74]. Duration of lockdown-induced detraining appears ambiguous, but studies show fitness levels typically recovered within 2–8 weeks postlockdown depending on the underlying fitness variables [54, 58]. With this in mind, HIIT can be implemented post-lockdown (first ~2 weeks of reopening) to rapidly enhance both intermittent and continuous aerobic capacity, particularly if sufficient training was conducted during the lockdown [75].

It remains unclear whether structured interventions or supervised training lead to greater retention of physical fitness and body composition than unstructured or unsupervised training. Scoz et al. [60] reported reduced eccentric strength of the knee flexors in a group without frequent training monitoring, while most other measures were largely preserved. In a non-lockdown study, both supervised and online home-training (both using small dumbbells) improved jumping ability and maximal upper/lower-body strength, but not 20 m sprint performance and body composition which remained unchanged [76]. Broadly, reduced training volume may hinder adaptations, and maintaining high intensity training could mitigate negative effects [2, 58, 67, 68]. Robust and adaptable athlete-support systems are necessary to optimise training and support during constrained situations or lockdowns [77]. Despite initial challenges and declines in performance, multiple world records in athletics were broken in 2020–21, suggesting athletes (i) may benefit from reduced competition and travel demands; (ii) regained fitness quickly or enhanced fitness qualities previously neglected; and/or (iii) maintained training through special arrangements [78, 79, 80]. In that regard, a substantial decline of performance was evident in 2020 among top-ranked swimmers [81] as well as track and field [82], but rebounded in 2021, even surpassing pre-COVID-19 levels [82].

### Effects on body composition

Changes in body composition varied in direction and magnitude across studies. Some individual studies reported increases [30, 57, 59], decreases [28, 61], or no change [27, 53, 55, 56, 60]. Collectively, our meta-analysis indicates non-significant *trivial* changes in body mass (ES = −0.115), which suggests that athletes generally maintained their weight despite lockdown-related disruptions. In contrast, the findings of an earlier meta-analysis [16] indicated an increase in body mass, and these contrasting outcomes likely relate to differences in the inclusion criteria. For example, Rosa et al. [16] included young participants (< 18 years old) where the reported increase in body mass was substantial, e.g., nearly 5 kg [83] (which could inflate the final value of all studies), whereas our study focused on adult athletes only. The impact of weight-class sports on body mass changes is not conclusive as one study reported an increase [59] and another no change [54]. Meta-regression indicates that body mass changes were most pronounced in Türkiye, while the UK was the least affected among the countries that were represented in the studies. The increase observed in Türkiye may be attributed to insufficient training prescriptions and/or dietary monitoring [30], whereas this was not observed in the UK [27].

Consistent with the findings on body mass, we also observed a lack of change in lean body mass across studies [54, 56, 57]; while the changes in fat mass appeared inconsistent, with one study reporting an increase [54] and others finding no significant alterations [56, 57, 61]. It is possible that alteration in muscle mass is likely due to the absence of sufficient strength training stimuli [61], while increases in fat mass or body fat percentage could be attributed to decreased training volume and/or lack of nutritional follow-up during lockdown [54]. Therefore, maintenance of fat mass could be associated with an individualised nutritional plan for athletes to follow during lockdown [56, 61]. It appears that the impact of lockdowns on body composition was highly variable and individual, and structured home-based training programs, such as those implemented by Anderson et al. [27], might be appropriate for maintaining body composition.

### Strengths and limitations

Our review’s strengths include its comprehensive coverage of the relevant literature, stricter inclusion criteria, careful evaluation of the risk of bias of the included studies, and assessment of their certainty of evidence. Another strength of our review is the use of leave-one-out sensitivity analyses, which confirmed the consistency of the results and enhanced confidence in the reliability of the pooled estimates. However, the high heterogeneity of the included studies is a limitation (likely due to differences in populations, protocols, or measurement methods), which was addressed partly through meta-regression and subgroup analyses. However, it is important to interpret the results of these analyses with caution, given their observational nature, which limits causal inferences [50]. Another key limitation is the overall very low certainty of evidence across all meta-analysed outcomes, which suggests that the confidence in the findings may be limited. Additionally, the presence of publication bias in studies related to body mass is another concern, as it may have led to an overestimation or underestimation of the true effects. Due to the limited number of studies, meta-analytical calculations were restricted to body mass, CMJ height, and CMJ relative peak power, preventing the ability to draw conclusions for other body composition or physical/fitness performance metrics. Lastly, only studies published in English were included in this review, and as a result, relevant studies published in other languages may have been excluded, and this shall be considered when appraising our results.

## CONCLUSIONS

Overall, the effects of the COVID-19 lockdown on athletes’ physical fitness and body composition were varied, with outcomes (as observed in most studies) ranging from noticeable impairments (e.g., endurance-related measures) to relative stability (e.g., body mass, CMJ jump height, maximum strength), while other outcomes showed mixed results (e.g., sprint performance). Our meta-analysis confirmed declines in CMJ relative peak power (albeit with a *trivial-small* effect). Collectively, these results suggest that while the lockdown had some impact, it was certainly not greater than *trivial-small* for the specific variables assessed in our meta-analysis (body mass, CMJ height, and CMJ relative peak power), though even small changes can be crucial for high-level athletes. To counteract the negative effects of detraining, targeted, individualised and sport-specific training countermeasures should be prioritised. These findings must be considered alongside certain limitations of the current analysis, particularly the overall very low certainty of evidence, which may impact confidence in the results.

### Practical Application

The outcome of this systematic review and meta-analysis on body composition and physical fitness showed that the effects of the COVID-19 lockdown were relatively mixed (impaired, maintained, or both). It is important that home-based training programs incorporating resistance and aerobic exercises (e.g., bodyweight-based exercises, simple tools) are designed to mitigate some of the potential negative declines in physical fitness. Individualised programs can integrate high-intensity training protocols as well as activities that effectively target specific performance outcomes (e.g., jumping, throwing, linear and non-linear sprints, deceleration, ball handling drills), provided that safety is maintained throughout. The use of a stationary bike, broad or personalised nutrition advice (which should be revisited regularly) may also be useful in assisting athletes during periods of isolation.

We also suggest providing a thorough support program for athletes during isolated or home training, e.g., alternative training, training monitoring, injury prevention, recovery strategy, sleep, mental health and wellbeing, as outlined elsewhere [78, 84, 85]. This approach should take into account the fact that Ramadan fasting during the COVID-19 lockdown may have been particularly more challenging for observant athletes [86, 87]. When returning to normal training after reduced or modified training (e.g., lockdown), a gradual increase in workload is advisable. In parallel, tailored approaches are required to manage health emergencies in sport settings (e.g., COVID-19, Mpox), supported by enhanced understanding, strategies, and preparedness [88]. We acknowledge the usefulness (and limitations) of AI conversational tools such as ChatGPT in providing recommendations on training, nutrition, and related areas [89, 90], particularly during lockdown situations. However, this topic is beyond the scope of the current review. Future research should investigate optimal home-based training interventions (including the use of AI conversational tools) to maintain or improve athlete performance during periods of restricted access to training facilities.

## Supplementary Material

Changes in physical fitness and body composition of athletes after the COVID-19 lockdown: a systematic review, meta-analysis, and meta-regression, with assessment of the certainty of evidence

## Data Availability

The datasets generated and analysed during the current meta-analysis are available from the corresponding author upon reasonable request.
