# Supplementary material for: Changes in physical fitness and body composition of athletes after the COVID-19 lockdown: a systematic review, meta-analysis, and meta-regression, with assessment of the certainty of evidence
Source: Biol Sport. 2025 Oct 1;43:463–88. doi: 10.5114/biolsport.2026.153307 (PMC12954494; doi:10.5114/biolsport.2026.153307)
Supplement: Changes in physical fitness and body composition of athletes after the COVID-19 lockdown: a systematic review, meta-analysis, and meta-regression, with assessment of the certainty of evidence [file JBS-43-56512-s1.pdf]

SUPPLEMENTARY MATERIALS

(A)

Study name	Point	Standard error	Variance	Lower limit	Upper limit	Z-Value	p-Value
Cohen, 2020	-0,031	0,106	0,011	-0,238	0,177	-0,292	0,770
Ambrozy, 2021	-0,084	0,085	0,007	-0,252	0,083	-0,986	0,324
Campa, 2021	-0,014	0,105	0,011	-0,221	0,192	-0,137	0,891
Fatih, 2021	-0,037	0,106	0,011	-0,245	0,171	-0,347	0,729
Sannicandro, 2021	0,048	0,065	0,004	-0,080	0,176	0,740	0,460
Spyrou, 2021	-0,026	0,104	0,011	-0,230	0,178	-0,254	0,800
Scoz, 2022 Team A	-0,017	0,109	0,012	-0,230	0,196	-0,158	0,874
Scoz, 2022 Team B	-0,007	0,106	0,011	-0,216	0,202	-0,066	0,947
Villaseca-Vicuna, 2022	-0,038	0,106	0,011	-0,246	0,169	-0,363	0,716
Tan, 2023	-0,029	0,105	0,011	-0,235	0,177	-0,273	0,785
Anderson, 2023	-0,037	0,106	0,011	-0,245	0,171	-0,352	0,725
	-0,025	0,097	0,009	-0,214	0,164	-0,257	0,797

Statistics with study removed

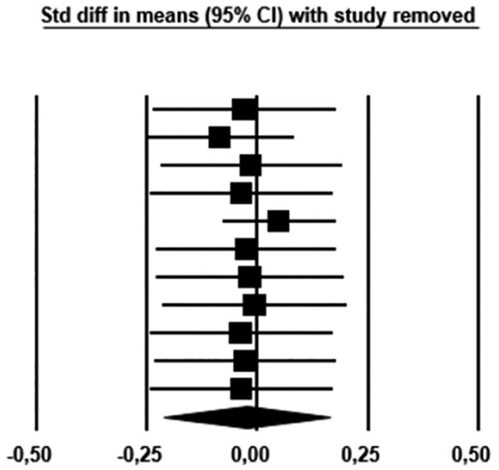

(B)

Study name	Point	Standard error	Variance	Lower limit	Upper limit	Z-Value	p-Value
Cohen, 2020	-0,327	0,209	0,044	-0,737	0,084	-1,561	0,119
Fatih, 2021	-0,318	0,212	0,045	-0,734	0,099	-1,496	0,135
Pedersen, 2021	-0,278	0,199	0,040	-0,668	0,112	-1,397	0,162
Rampinini, 2021	-0,362	0,205	0,042	-0,763	0,039	-1,769	0,077
Sannicandro, 2021	-0,148	0,132	0,018	-0,407	0,111	-1,118	0,264
Valenzuela, 2021	-0,298	0,200	0,040	-0,690	0,094	-1,488	0,137
Spyrou, 2021	-0,299	0,203	0,041	-0,697	0,099	-1,474	0,140
Villaseca-Vicuna, 2022	-0,343	0,210	0,044	-0,755	0,068	-1,635	0,102
Tan, 2023	-0,282	0,202	0,041	-0,678	0,114	-1,394	0,163
Anderson, 2023	-0,415	0,170	0,029	-0,748	-0,082	-2,443	0,015
	-0,308	0,185	0,034	-0,671	0,056	-1,659	0,097

Statistics with study removed

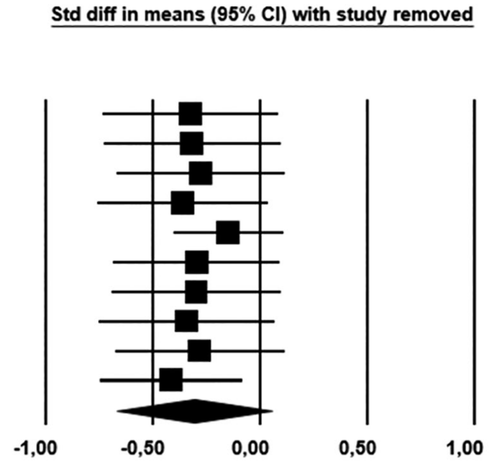

(C)

Study name	Point	Standard error	Variance	Lower limit	Upper limit	Z-Value	p-Value
Cohen, 2020	-0,167	0,093	0,009	-0,349	0,014	-1,805	0,071
Fatih, 2021	-0,208	0,096	0,009	-0,396	-0,020	-2,170	0,030
Pedersen, 2021	-0,188	0,089	0,008	-0,362	-0,014	-2,116	0,034
Rampinini, 2021	-0,195	0,095	0,009	-0,381	-0,008	-2,048	0,041
Spyrou, 2021	-0,228	0,089	0,008	-0,403	-0,052	-2,544	0,011
Tan, 2023	-0,197	0,091	0,008	-0,375	-0,018	-2,157	0,031
	-0,197	0,084	0,007	-0,362	-0,032	-2,346	0,019

Statistics with study removed

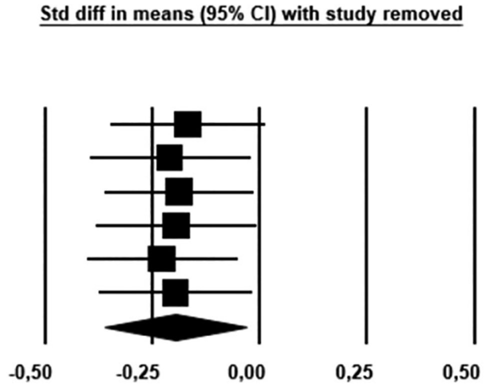

FIG. S1. Leave-one-out sensitivity analysis for body mass (A), CMJ height (B), and CMJ relative peak power (C) after the COVID-19 lockdown.

TABLE S1. Quality assessment of the remaining included studies in the systematic review using the JBI Critical Appraisal Checklist.

	1	2	3	4	5	6	7	8	9	RoB (%)
Horizontal jump										
Ambrozy et al. [59]	Y	Y	Y	NA	N	Y	Y	U	Y	75
Fatih et al. [30]	U	Y	Y	NA	N	Y	Y	Y	Y	75
Spyrou et al. [56]	Y	Y	Y	NA	N	Y	Y	Y	Y	88
Global risk of bias score (%)										79
Linear sprint performance										
Ambrozy et al. [59]	Y	Y	Y	NA	N	Y	Y	U	Y	75
Fatih et al. [30]	U	Y	Y	NA	N	Y	Y	Y	Y	75
Sannicandro & Bisciotti [28]	Y	Y	Y	NA	N	Y	Y	U	Y	75
Spyrou et al. [56]	Y	Y	Y	NA	N	Y	Y	Y	Y	88
Pedersen et al. [31]	Y	Y	Y	NA	N	Y	Y	Y	Y	88
Villaseca-Vicuña et al. [57]	Y	Y	Y	NA	N	Y	Y	Y	Y	88
Global risk of bias score (%)										81
Maximum strength										
Anderson et al. [27]	Y	Y	Y	NA	N	Y	Y	Y	Y	88
Ambrozy et al. [59]	Y	Y	Y	NA	N	Y	Y	U	Y	75
Villaseca-Vicuña et al. [57]	Y	Y	Y	NA	N	Y	Y	Y	Y	88
Pedersen et al. [31]	Y	Y	Y	NA	N	Y	Y	Y	Y	88
Valenzuela et al. [58]	Y	Y	Y	NA	Y	Y	Y	Y	Y	100
Bataltha et al. [29]	Y	Y	Y	NA	N	Y	Y	Y	Y	88
Fatih et al. [30]	U	Y	Y	NA	N	Y	Y	Y	Y	75
Scoz et al. A [60]	Y	Y	Y	NA	Y	Y	Y	Y	Y	100
Scoz et al. B [60]	Y	Y	Y	NA	Y	Y	Y	Y	Y	100
Global risk of bias score (%)										89
Muscular endurance										
Ambrozy et al. [59]	Y	Y	Y	NA	N	Y	Y	U	Y	75
Fatih et al. [30]	U	Y	Y	NA	N	Y	Y	Y	Y	75
Global risk of bias score (%)										75
Aerobic-related										
Ambrozy et al. [59]	Y	Y	Y	NA	N	Y	Y	Y	Y	88
Anderson et al. [27]	Y	Y	Y	NA	N	Y	Y	Y	Y	88
Fatih et al. [30]	Y	Y	Y	NA	N	Y	Y	Y	Y	88
Sannicandro & Bisciotti [28]	Y	Y	Y	NA	N	Y	Y	Y	Y	88
Rampinini et al. [15]	Y	Y	Y	NA	N	U	Y	Y	Y	75
Villaseca-Vicuña et al. [57]	Y	Y	Y	NA	N	Y	Y	Y	Y	88
Global risk of bias score (%)										85
Flexibility										
Ambrozy et al. [59]	Y	Y	Y	NA	N	Y	Y	U	Y	75
Fatih et al. [30]	Y	Y	Y	NA	N	Y	Y	Y	Y	88
Bataltha et al. [29]	Y	Y	Y	NA	N	Y	Y	U	Y	75
Global risk of bias score (%)										79
Muscle mass / lean body mass										
Spyrou et al. [56]	Y	Y	Y	NA	N	Y	Y	Y	Y	88
Tan et al. [54]	Y	Y	Y	NA	Y	Y	Y	Y	Y	100
Villaseca-Vicuña et al. [57]	Y	Y	Y	NA	N	Y	Y	Y	Y	88
Global risk of bias score (%)										92
Fat mass / fat percentage										
Campa et al. [61]	Y	Y	Y	NA	N	Y	Y	Y	Y	88
Spyrou et al. [56]	Y	Y	Y	NA	N	Y	Y	Y	Y	88
Villaseca-Vicuña et al. [57]	Y	Y	Y	NA	N	Y	Y	Y	Y	88
Global risk of bias score (%)										88
Skinfold thickness										
Andersen et al. [27]	Y	Y	Y	NA	N	Y	Y	Y	Y	88
Villaseca-Vicuña et al. [57]	Y	Y	Y	NA	N	Y	Y	Y	Y	88
Global risk of bias score (%)										88

Note; Y: Yes; N: No; U: Unclear; NA: Not Applicable; RoB: Risk of Bias; Item 1: Is it clear in the study what is the 'cause' and what is the 'effect' (i.e. there is no confusion about which variable comes first)?; Item 2: Were the participants included in any comparisons similar?; Item 3: Were the participants included in any comparisons receiving similar treatment/care, other than the exposure or intervention of interest?; Item 4: Was there a control group?; Item 5: Were there multiple measurements of the outcome both pre and post the intervention/exposure?; Item 6: Was follow up complete and if not, were differences between groups in terms of their follow up adequately described and analysed?; Item 7: Were the outcomes of participants included in any comparisons measured in the same way?; Item 8: Were outcomes measured in a reliable way?; Item 9: Was appropriate statistical analysis used?;

TABLE S2. Summary of included studies on the effects of COVID-19 lockdown on physical performance and body composition. The *P* value and effect size statistics Cohen's d are shown. ↑ increase, ↓ decrease, ↔ no significant change.

Authors and Country	Participant Details	Lockdown duration / Test dates	Intervention (training characteristics or status)	Measurements / testing protocols	Changes in performance and body composition (pre to post)	Conclusion
Ambrozy et al. [59] Poland	Kickboxing (N=20); International level; Male; Age 25.2 ± 3.02 y; Stature 181 ± 4 cm; Mass 82.5 ± 4.89 kg	Lockdown: ~6 weeks Pre-test: 13 March 2020 Post-test: 21 May 2020	– Training prescription: Yes – Training site: Home – Supervision: Yes – Frequency (per week): 6 – HIIT: NA – Dietary monitoring/ guidelines: Yes *Remarks:* training frequency and hours are the same as pre-lockdown	– Basic anthropometric (body mass, stature) – Aerobic capacity test ($\dot{V}O_{2max}$) – 50 m sprint – Standing long jump – 1000 m run – Grip strength – Pull up – 5 × 10 m shuttle run – 30-s sit-ups – Forward bend	Body mass 82.50 ± 4.89 → 85.15 ± 4.87 (P < **0.001**, d=0.54) BMI (kg/m^2) 25.27 ± 1.09 → 26.08 ± 1.13 (P < **0.001**, d=0.75) 50 m sprint 8.25 ± 0.30 → 8.41 ± 0.33 (P=0.097, d=0.50) SLJ (cm) 208 ± 16 → 203 ± 18 (P < **0.001**, d=0.36) 1000 m (min) 3.91 ± 0.33 → 4.06 ± 0.26 (***P*=0.028**, d=0.45) R hand grip (kg) 55.97 ± 2.07 → 55.72 ± 1.98 (P=0.077, d=0.12) L hand grip (kg) 55.09 ± 2.09 → 55.05 ± 2.08 (P=0.642, d=0.02) Pull up (reps) 19.7 ± 4.1 → 17.2 ± 4.2 (P < **0.001**, d=0.61) 5 × 10 m shuttle run (s) 10.84 ± 0.69 → 11.00 ± 0.75 (P=**0.015**, d=0.23) Forward bend (cm) 7.25 ± 1.25 → 5.55 ± 1.10 (P < **0.001**, d=1.36) 30 s sit-up (reps) 27.55 ± 3.91 → 27.20 ± 4.19 (P=0.349, d=0.09) $\dot{V}O_{2max}$ (mL/kg/min) 47.2 ± 3.7 → 45.6 ± 3.5 (P=**0.001**, d=0.50)	↑ Body mass. ↓ Athletic performance and physical fitness.
Anderson et al. [27] United Kingdom	Football (N=20); Elite professional players; Male; Age 26 ± 4 y; Stature 182 ± 6 cm; Mass 79.2 ± 7.1 kg.	Lockdown: 94 days (~13 weeks) Pre-test: 3 days before lockdown. Post-test: the week after lockdown.	– Training prescription: Yes – Supervision: Yes – Training side: Home; limited outdoors. – Frequency (per week): 6–7 – HIIT: Yes – Dietary monitoring/ guidelines: Yes *Remarks:* periodised training (4–5 aerobic/~2 strength), progressed in 3 distinct phases to achieve specific objectives in muscular and aerobic developments; more physical/fitness focus; lack of specific training.	– Body composition – CMJ – Eccentric hamstring strength – Submaximal 30–15 intermittent fitness test	Body mass (kg) 79.3 ± 6.7 → 80.0 ± 7.3 (P=0.13, d= 0.11) Skinfolds, Σ6 (mm) 54.1 ± 14.8 → 56.7 ± 15.2 (P=0.07, d=0.18) CMJ height (cm) 38.4 ± 3.4 → 40.9 ± 4.1 (P= 0.06, d=-0.67) ECC hamstring strength (N) 1035 ± 158 → 1009 ± 140 (P=0.46, d=0.18) IFT % of max HR (%) 81.3 ± 5.2 → 82.3 ± 7.3 (P=0.63, d=0.15)	↔ Body composition. ↔Athletic performance and physical fitness. Alternative training (home based, no specific training) e.g., HIIT and resistance exercises preserved fitness levels.

TABLE S2. Continue.

Authors and Country	Participant Details	Lockdown duration / Test dates	Intervention (training characteristics or status)	Measurements / testing protocols	Changes in performance and body composition (pre to post)	Conclusion
Batalha et al. [29]<br>Portugal	Handball (N=16);<br>Male;<br>National 2nd division;<br>Age 22.38 y;<br>Stature 1.77 ± 0.38 m;<br>Mass 83.4 ± 15.6 kg.	Lockdown: 10 weeks (70 days)<br>Pre-test: 18–21 January<br>Post-test: week of 12 April	– Training prescription: Yes<br>– Training site: Home<br>– Supervision: NA<br>– Frequency (per week): 3<br>– HIIT: NA<br>– Dietary monitoring/ guidelines: NA<br>*Remarks:* players performed 45 min of aerobic training (cycling or running) and 30 min of strength training (core training, arm, and squats).	– Isokinetic strength (peak torque)<br>– Shoulder ROM<br>– Ball release velocity	*At 60°/s*<br>D-shoulder ER, peak torque (Nm) 41 ± 7 → 35 ± 6 (P=**0.001**, d=1.04)<br>ND-shoulder ER, peak torque (Nm) 38 ± 6 → 34 ± 7 (P=**0.008**, d=0.60)<br>ND-shoulder IR, peak torque (Nm) 48 ± 7 → 43 ± 7 (P **< 0.001**, d=1.77)<br>*At 180°/s*<br>D-shoulder ER, peak torque (Nm) 38 ± 7 → 34 ± 7 (P < **0.001**, d=1.40)<br>D ER fatigue index (%) 22 ± 8 → 28 ± 7 (P < **0.001**, d=1.11)<br>D IR fatigue index (%) 18 ± 8 → 26 ± 10 (P=**0.001**, d=0.92)<br>ND-shoulder ER, peak torque (Nm) 36 ± 7 → 32 ± 7 (P=**0.002**, d=0.91)<br>ROM D-IR (º) 63 ± 9 → 53 ± 10 (P=**0.001**, d=1.10)<br>ROM D-ER (º) 78 ± 13 → 75 ± 14 (P=0.231, d=0.31)<br>ROM ND-IR (º) 61 ± 10 → 59 ± 10 (P=0.547, d=0.154)<br>ROM ND-ER (º) 78 ± 11 → 75 ± 10 (P=0.088, d=0.36)<br>Jump shot ball release (m/s) 22 ± 1 → 20 ± 3 (P=**0.011**, d=0.73)	↓ Shoulder (rotators) strength and motion range; and a decline in ball release velocity<br>Specific training program is necessary to counteract detraining effects in athletes
Campa et al. [61]<br>Italy	Football (N=15);<br>Italian First division (Serie A) team;<br>Male;<br>Age 30.5 ± 3.6 y;<br>Mass 79.6 ± 7.6 kg;<br>Stature 1.82 ± 0.1 m.	Lockdown: ~14 weeks (February to May)<br>Pre-test: end of February 2020<br>Post-test: end of May 2020	– Training prescription: Yes<br>– Training site: Home<br>– Supervision: NA<br>– Frequency (per week): 6<br>– HIIT: No<br>– Dietary monitoring/ guidelines: Yes<br>*Remarks:* individualised nutritional and supplementation plan; 84 sessions (14 weeks) all home-based with 3 sessions/week of aerobic drills aerobic (mainly running/ cycling-based MIIT) training and strength-related stimuli. One session/week of aerobic drills (mainly running/cycling-based LIT).	– Anthropometric<br>– Body composition (fat mass and muscle mass)<br>– Bioelectrical Impedance Vector Analysis<br>– Phase Angle	Body mass (kg) 79.6 ± 7.6 → 78.6 ± 7.8 (P < **0.05**, d=−0.72)<br>BMI (kg/m^2) 23.7 ± 1.0 → 23.4 ± 1.1 (P < **0.05**, d=−0.73)<br>R/H (Ohm/m) 258.9 ± 22.4 → 263.9 ± 23.5 (P < **0.05**, d=0.66)<br>Xc/H (Ohm/m) 35.6 ± 3.6 → 34.6 ± 3.5 (P < **0.05**, d=−0.85)<br>PhA (°) 8.0 ± 0.5 → 7.5 ± 0.5 (P < **0.05**, d=−1.56)<br>Fat mass (%) 14.1 ± 1.7 → 14.3 ± 1.9 (P > 0.05, d=0.13]<br>Muscle mass (kg) 28.2 ± 2.3 → 27.4 ± 2.7 (P < **0.05**, d=−2.04)	↔ Fat mass.<br>↓ Body mass, muscle mass and phase angle. Very large reduction in muscle mass, likely as a result of inadequate training stimulus.

TABLE S2. Continue.

Authors and Country	Participant Details	Lockdown duration / Test dates	Intervention (training characteristics or status)	Measurements / testing protocols	Changes in performance and body composition (pre to post)	Conclusion
Cohen et al. [55] Colombia	Football (N=16); America de Cali Football Team; Male; Age 24.3 ± 3.8 y; Stature 1.79 ± 0.07 m.	Lockdown: March 20–April 2 (15 weeks) Pre-test: February 20 and March 6, 2020 Post-test: end of June 2020	– Training prescription: Yes<br>– Training site: Home<br>– Supervision: NA<br>– Frequency (per week): 5–8<br>– HIIT: NA<br>– Dietary monitoring/ guidelines: NA<br>*Remarks:* isolated circuit resistance and aerobic interval training, 5 × /week (March-April). Then, individual training plans (circuit training, bodyweight and resistance bands (CRT) (6 April – 7 June), 5 × / week. Solo home CRT (6 × /week); intermittent aerobic interval training with and without football (2 × /week) (10–30 June).	– Basic anthropometry<br>– CMJ	Body mass (kg) 79.2 ± 9.0 → 79.6 ± 9.4 (P=0.52, d=0.05)<br>CMJ height (cm) 39.93 ± 5.12 → 39.13 ± 5.02 (P=0.34, d=−0.16)<br>RSImod (au) 0.49 ± 0.07 → 0.47 ± 0.08 (P=0.22, d=−0.26)<br>ECC peak velocity (m/s) 1.45 ± 0.27 → −1.40 ± 0.24 (P=0.26, d=−0.19)<br>ECC peak power (W/kg) −20.0 ± 4.8 → −18.7 ± 4.2 (P=0.12, d=−0.30)<br>CON peak velocity (m/s) 2.92 ± 0.18 → 2.89 ± 0.17 (P=0.41, d=−0.15)<br>CON peak power (W/kg) 56.4 ± 6.2 → 54.4 ± 5.7 (P=0.06, d=−0.33)<br>Landing peak force (N/kg) 76 ± 26 → 66 ± 24 (P=**0.02**, d=0.40)	↔ Body mass.<br>↔ Athletic performance and physical fitness (jump height, RSImod, peak concentric and eccentric power).
Fatih et al. [30] Türkiye	Football (N=20); Amateur players; Male; Age 19.05 ± 1.31 y; Stature 175 ± 7 cm.	Lockdown: 80 days Pre-test: Mid-March 2020 Post-test: first week of June 2020	– Training prescription: No<br>– Training site: NA<br>– Supervision: NA<br>– Frequency (per week): NA<br>– HIIT: NA<br>– Dietary monitoring/ guidelines: NA	– Anthropometry<br>– Flexibility (Sit-and-reach)<br>– Balance (Flamingo)<br>– Illinois agility<br>– 30 m sprint<br>– Strength / endurance / power (push up, 30 s sit up, standing long jump, handgrip strength, medicine ball throw)<br>– Aerobic (Yoyo-IRL1)<br>– CMJ	Body mass (kg) 68.74 ± 8.14 → 69.45 ± 8.66 (P=**0.005**, d=0.62)<br>BMI (kg/m^2) 22.35 ± 1.81 → 22.58 ± 1.97 (P=**0.006**, d=0.61)<br>Sit and reach (cm) 39.15 ± 5.56 → 38.72 ± 5.06 (P=**0.028**, d=0.48)<br>HGS (kg) 46.51 ± 8.53 → 45.82 ± 8.63 (P=**0.036**, d=0.46)<br>SLJ (cm) 227 ± 25 → 224 ± 23 (P=0.112)<br>MBT (cm) 1054 ± 250 → 1029 ± 237 (P=**0.025**, d=0.50)<br>Flamingo balance (#) 3.9 ± 1.9 → 4.2 ± 1.6 (P=0.197)<br>30 m sprint (s) 4.28 ± 0.28 → 4.51 ± 0.37 (P=**0.000**, d=0.86)<br>Illinois agility (s) 15.99 ± 0.79 → 16.46 ± 0.76 (P=**0.000**, d=0.84)<br>30 s sit-up (reps) 28.2 ± 7.7 → 26.6 ± 6.8 (P=**0.003**, d=0.66)<br>30 s push-up (reps) 29.4 ± 6.9 → 27.7 ± 5.9 (P=**0.001**, d=0.74)<br>CMJ height (cm) 44.17 ± 5.48 → 42.87 ± 5.17 (P=**0.000**, d=0.83)<br>CMJ peak power (W) 3740 ± 519 → 3694 ± 509 (P=**0.001**, d=0.77)<br>Yoyo-IRL1 (m) 2313 ± 541 → 1977 ± 425 (P=**0.000**, d=0.87)<br>$\dot{V}O_{2max}$ (mL/kg/min) 55.8 ± 4.5 → 53.0 ± 3.6 (P=**0.000**, d=0.87)	↓ Body mass.<br>↓ Athletic performance and physical fitness.<br>Long-term detraining period (80 days) caused negative effects in all parameters; greater in sprint, agility, vertical jump, and aerobic performances.

TABLE S2. Continue.

Authors and Country	Participant Details	Lockdown duration / Test dates	Intervention (training characteristics or status)	Measurements / testing protocols	Changes in performance and body composition (pre to post)	Conclusion
Pedersen et al. [31] Norway	Football (N=9); Second division players; Female; Age 18.8 ± 1.9 y; Mass 61.3 ± 3.7 kg; Stature 1.68 ± 0.4 m.	Lockdown: ~3 months Pre-test: 1 week prior. Post-test: at the end of lockdown.	– Training prescription: Yes – Training site: Home – Supervision: NA – Frequency (per week): NA – HIIT: NA (not explicit) – Dietary monitoring/ guidelines: NA *Remarks:* Home-based and group-based training, 233 min/week (no gym facilities or normal football play). Type: strength, jump, and sprint exercises (greater time spent compared to regular pre-season training).	– 1RM partial squat – CMJ –15 m sprint	1RM partial SQ (kg) 104 ± 12 → 101 ± 11 (P=0.28) Relative 1RM-SQ (kg/w) 1.69 ± 0.24 → 1.65 ± 0.23 (P=0.39) CMJ height (cm) 28.1 ± 2.3 → 26.8 ± 1.9 (P=0.09) CMJ power (W) 2665 ± 167 → 2605 ± 109 (P=0.40) 5 m sprint (s) 1.03 ± 0.04 → 1.04 ± 0.05 (P=0.52) 10 m sprint (s) 1.86 ± 0.05 → 1.88 ± 0.05 (P=0.28) 15 m sprint (s) 2.60 ± 0.08 → 2.61 ± 0.07 (P=0.52)	↔ Athletic performance and physical fitness. Both home-based and group-based interventions successfully maintained strength, jumping, and sprinting abilities
Rampinini et al. [15] Italy	Football (N=21); Italian Serie A club; Male; Age 25.4 ± 5.0 y; Mass 78.5 ± 5.7 kg; Stature 1.82 ± 0.05 m.	Lockdown: 13 weeks (9 March to 3 May) Pre-test: February 2020 Post-test: May 2020	– Training prescription: Yes – Training site: Home – Supervision: NA – Frequency (per week): 8 – HIIT: Yes – Dietary monitoring/ guidelines: NA *Remarks:* home-based, 4–5 aerobic sessions (medium to high intensity) using in-home stationary equipment (treadmill or bike); 2–3 strength training sessions (bodyweight and small weights). Weekly training volume was ~380 min.	– Mognoni test (aerobic fitness): 6-min continuous run at 13.5 km/h (total 1350 m) – CMJ	Mognoni test, NA (significant improvement post-lockdown [lower blood lactate levels indicating better aerobic fitness]). CMJ height (cm) 51.4 ± 5 → 52.1 ± 4.7 ($P > 0.05$) CMJ peak power (W/kg) 62.0 ± 8.9 → 60.3 ± 7.0 ($P < 0.05$) *Data not explicitly provided (estimated from figures)*	↑ Aerobic fitness. ↓ Anaerobic power (↔ jump heights).
Sannicandro and Bisciotti [28] Italy	Football (N=26); Male; Age 26.07 ± 3.96 y; Mass 76.9 ± 2.51 kg; Stature 181 ± 4 cm;	Lockdown: 11 March to 4 May Pre-test: February 26–29, 2020 Post-test: May 6–9, 2020	– Training prescription: Yes – Training site: Home – Supervision: No – Frequency (per week): NA – HIIT: NA – Dietary monitoring/ guidelines: No	– Basic anthropometric – Vertical jump – Monopodalic hop – 10 m sprint – Incremental test for VMA and $\dot{V}O_{2max}$	Body mass (kg) 76.8 ± 2.5 → 75.0 ± 2.3 (P=**0.001**, d=0.69) BMI (kg/m^2) 23.3 ± 0.8 → 22.8 ± 0.7 (P=**0.0005**, d=0.67) CMJ height (cm) 46.7 ± 3.9 → 40.8 ± 3.2 (P=**0.0005**, d=1.66) SJ (cm) 44.0 ± 3.2 → 39.6 ± 2.1 (P=**0.0005**, d=1.68) Léger test (min) 12.58 ± 1.20 → 10.42 ± 1.02 (P=**0.0005**, d=1.92) VMA (km/h)14.4 ± 0.6 → 13.2 ± 0.5 (P=**0.0005**, d=1.99) $\dot{V}O_{2max}$ (mL/kg/min) 50.8 ± 4.6 → 45.6 ± 4.8 (P=**0.0005**, d=1.78) 10 m sprint (s) 1.91 ± 0.07 → 2.09 ± 0.05 (P=**0.0005**, d=2.99) Hop Left (cm) 195.4 ± 12.2 → 176.4 ± 11.0 (P=**0.0005**, d=1.64) Hop Right (cm) 198.1 ± 11.8 → 178.6 ± 15.8 (P=**0.0005**, d=1.42)	↓ Body mass. ↓ Athletic performance and physical fitness levels. Home training appeared insufficient to maintain adaptations achieved from the regular season.

TABLE S2. Continue.

Authors and Country	Participant Details	Lockdown duration / Test dates	Intervention (training characteristics or status)	Measurements / testing protocols	Changes in performance and body composition (pre to post)	Conclusion
Scoz et al. [60] Brazil	Football; Professional elite players; Team A (N=26) Age 26.15 ± 5.12 y; Stature 181 ± 7 cm. Team B (N=23) Age 26.47 ± 5.34 y; Stature 181 ± 7 cm.	Lockdown: 6 weeks *Note: has been confirmed within the criteria but exact dates not provided.*	*Team A* – Training prescription: Yes – Training site: Home – Supervision: Yes – Frequency (per week): Not explicit (2 × /day, daily) – HIIT: NA – Dietary monitoring/ guidelines: Yes *Remarks:* video calls 1/day (athlete-trainer). Nutritionist prescribed diets. Training with free-weight-bearing, calisthenics activities (dumbbells, kettlebells, and ankle weights); an hour each session. General training plan: strength exercises (40%), power exercises (20%), and aerobic conditioning (40%). *Team B* – Training prescription: Yes – Training site: Home – Supervision: Yes (limited) – Frequency (per week): Not explicit (2 × /day, daily) – HIIT: NA – Dietary monitoring/ guidelines: Yes *Remarks:* received an early instruction by team athletic trainer. Followed the same strategy/prescription/ equipment as team A, but not regularly monitored by trainer.	– Basic anthropometry – Isokinetic test procedure	*Team A* (60°/s) Body mass (kg) 77.03 ± 7.99 → 76.19 ± 6.71 ($P > 0.05$) BMI (kg/m^2) 23.39 ± 0.17 → 23.14 ± 0.14 ($P > 0.05$) Knee extensors CON PT (N) 242.70 ± 43.21 → 244.04 ± 38.09 (P=0.84) ECC PT (N) 304.41 ± 47.29 → 302.04 ± 54.82 (P=0.74) Knee flexors CON PT (N) 142.62 ± 25.30 → 154.04 ± 27.01 (P=0.17) ECC PT (N) 179.79 ± 27.06 → 175.91 ± 31.06 (P=0.39) HQ Ratios Conventional 0.59 ± 0.06 → 0.63 ± 0.05 (P=0.91) Functional 0.74 ± 0.03 → 0.72 ± 0.04 (P=0.72) *Team B* (60°/s) Body mass (kg) 76.08 ± 8.43 → 74.55 ± 7.93 ($P > 0.05$) BMI (kg/m^2) 23.33 ± 0.19 → 22.86 ± 0.18 ($P > 0.05$) Knee extensors CON PT (N) 241.91 ± 46.05 → 238.29 ± 47.35 (P=0.79) ECC PT (N) 294.91 ± 61.55 → 293.04 ± 44.11 (P=0.93) Knee flexors CON PT (N) 148.75 ± 24.91 → 144.75 ± 24.34 (P=0.57) ECC PT (N) 294.37 ± 61.60 → 185.75 ± 27.10 ($P <$ **0.01**, d= 2.31) HQ Ratios Conventional 0.61 ± 0.12 → 0.61 ± 0.05 (P=0.81) Functional 1.22 ± 0.36 → 0.78 ± 0.10 ($P <$ **0.01**, d=3.44)	↔ Body mass. ↓ Eccentric strength of knee flexors (Team B), i.e., group that did not receive frequent monitoring of daily training.
Spyrou et al. [56] Spain	Futsal (N=10); Elite players; Male; Age 26.7 ± 3.1 y; Mass 76.0 ± 6.6 kg; Stature 1.78 ± 0.06 m.	Lockdown: March 14, 2020 (70 days) Pre-test: 2–3 March 2020 Post-test: 12–13 May 2020	– Training prescription: Yes – Training site: Home – Supervision: Yes – Frequency (per week): 4–6 – HIIT: NA – Dietary monitoring/ guidelines: Yes *Remarks:* individualised nutritional plan (adjusted every 2 weeks). Bodyweight-based exercises (vertical and horizontal jumps, half and full squats, lunges, push-ups etc.), 2–3 × / week, 2–3 sets of 6–8 reps (for jumps) and 10–12 reps (squats) repetitions.	– Body composition – Vertical jump – Horizontal jump – Sprint acceleration	Body mass (kg) 76.0 ± 6.6 → 76.0 ± 6.7 (P=0.992, d=0.00) Fat mass (%) 9.29 ± 1.46 → 9.28 ± 1.70 (P=0.947, d=−0.02) LBM (kg) 50.1 ± 1.56 → 50.0 ± 1.48 (P=0.734, d=−0.11) CMJ height (cm) 35.8 ± 4.6 → 34.1 ± 3.5 (P=0.076, d=−0.63) CMJ peak power (W/kg) 53.3 ± 6.1 → 53.5 ± 6.7 (P=0.911, d=0.03) Horizontal jump, NA (figure) (P=0.243, d=−0.39) Sprint acceleration, NA (figure; poorer) (P=**0.004**, d=1.31) **Some data not explicitly provided*	↔ Body composition. ↔ Vertical and horizontal jumps. ↓ Sprint performance and specific CMJ kinetics (eccentric and landing phase).

TABLE S2. Continue.

Authors and Country	Participant Details	Lockdown duration / Test dates	Intervention (training characteristics or status)	Measurements / testing protocols	Changes in performance and body composition (pre to post)	Conclusion
Tan et al. [54] Malaysia	Wushu (N=13); Elite athletes; Male=7, Female=6 Age 21.8 ± 2.6 y; Stature 1.63 ± 0.07 m.	Lockdown: 18 March (16 weeks) Pre-test: 16 March Post-test: 13 July	– Training prescription: Yes<br>– Training site: Home<br>– Supervision: Yes<br>– Frequency (per week): 3<br>– HIIT: No<br>– Dietary monitoring/ guidelines: No<br>*Remarks:* multi-joint bodyweight resistance training using household items; variations of squats, lunges, push up, etc. (4–5 sets of 10–12 reps each); with additional loads using water bottles, gallon jugs, and heavy books when possible (not standardised).	– Body composition<br>– CMJ kinetics and kinematics	Body mass (kg) 59.25 ± 8.90 → 59.50 ± 8.50 ($P > 0.05$)<br>LBM (kg) 50.03 ± 9.93 → 48.52 ± 8.38 ($P > 0.05$)<br>Fat mass (kg) 9.82 ± 3.89 → 11.03 ± 3.51 ($P <$ **0.05**)<br>Body fat (%) 16.40 ± 6.97 → 18.70 ± 5.96 ($P <$ **0.05**)<br>CMJ height (cm) 38.98 ± 6.55 → 35.33 ± 6.79 ($P <$ **0.05**)<br>CMJ peak power (W/kg) 3324 ± 737 → 3196 ± 686 ($P <$ **0.05**)<br>CMJ peak velocity (m/s) 2.99 ± 0.22 → 2.90 ± 0.20 ($P <$ **0.05**)<br>CMJ RSImod (au) 0.49 ± 0.11 → 0.43 ± 0.12 ($P <$ **0.05**)	↓ Body composition (increased fat).<br>↓ Athletic performance and physical fitness (selected kinetics and kinematics of CMJ).
Villaseca-Vicuna et al. [57] Chile	Football (N=19); Elite players (Chilean national team at world cup); Female; Age 27 ± 4.19 y; Stature 162 ± 6 cm.	Lockdown: March 11 – July 22, 2020 (~150 days; 5 months) Pre-test: February 2020 (last week) Post-test: August 2020 (first week)	– Training prescription: Yes<br>– Training site: Home<br>– Supervision: Yes (3 × / week)<br>– Frequency (per week): 6<br>– HIIT: No<br>– Dietary monitoring/ guidelines: No<br>*Remarks:* Daily strength training, either *lower limb* focus (squat, deadlift, hip thrust), or *upper body* (push and pull exercises); or core exercises (bodyweights) for ~30 min (2 × /week each). Plus either functional activities (4 × /week) or intermittent ~20 m runs (2 × /week) at individualised velocity.	– Anthropometric<br>– Body composition<br>– Squat loading (MPV with 20–40 kg)<br>– 1RM squat<br>– 30 m sprint<br>– CMJ<br>– Kicking assessment.<br>– Yoyo-IRTL1	Body mass (kg) 58.38 ± 6.29 → 59.05 ± 6.19 (P=**0.014**, d=0.10)<br>Skinfolds, Σ6 (mm) 63.4 ± 13.4 → 66.4 ± 16.7 (P=0.310, d=0.14)<br>LBM (kg) 47.03 ± 2.56 → 46.43 ± 3.13 (P=0.265, d=−0.25)<br>Fat mass (%) 25.99 ± 3.04 → 26.69 ± 3.83 (P=0.211, d=0.18)<br>MPV20 (m/s) 1.18 ± 0.12 → 1.17 ± 0.12 (P=0.695, d=−0.08)<br>MPV30 (m/s) 1.07 ± 0.10 → 1.04 ± 0.12 (P=0.095, d=−0.37)<br>MPV40 (m/s) 0.95 ± 0.09 → 0.92 ± 0.11 (P=**0.041**, d−=0.34)<br>1RM-SQ (kg) 94.61 ± 26.84 → 89.37 ± 26.83 (P=0.107, d=−0.24)<br>Relative 1RM-SQ (kg/kg) 1.62 ± 0.42 → 1.51 ± 0.41 (P=0.056, d=−0.31)<br>CMJ height (cm) 28.71 ± 4.22 → 28.59 ± 4.84 (P=0.762, d=−0.04)<br>10 m sprint (s) 1.91 ± 0.07 → 1.89 ± 0.07 s (P=0.203, d=−0.18)<br>30 m sprint (s) 4.71 ± 0.13 → 4.69 ± 0.20 (P=0.107, d=−0.44)<br>Kicking (km/h) 79.89 ± 4.92 → 85.22 ± 5.04 ($P <$ **0.001**, d=1.02)<br>Yoyo-IRTL1 (m) 1,355 ± 347 → 1,211 ± 326 (P=**0.014**, d=−0.37)	↔ Body composition (but body mass increased).<br>↑ Kicking velocity.<br>↔ Athletic performance and physical fitness (strength, vertical jump, sprint).<br>↓ Intermittent endurance capacity.

TABLE S2. Continue.

Authors and Country	Participant Details	Lockdown duration / Test dates	Intervention (training characteristics or status)	Measurements / testing protocols	Changes in performance and body composition (pre to post)	Conclusion
Valenzuela et al. [58] 'Spain'	Badminton (N=7); Elite players (2 women and 5 men); Age 21.6 ± 3.14 y; Mass NA Stature NA	Lockdown: 7–10 weeks Pre-test: 2 weeks before lockdown started Post-test: 1 week after reopening	– Training prescription: Yes – Training site: NA – Supervision: Yes – Frequency (per week): ~8–12 – HIIT: Yes – Dietary monitoring/ guidelines: No *Remarks:* ~8–12 × /week (60–70 min). Included HIIT (badminton-specific tasks when possible) and RT circuits (using elastic bands and light to moderate weights). Only athlete 1 had access to a cycle ergometer, a hypoxia generator, BFR bands, heavy weights, and inertial systems.	– CMJ. – 1RM Squat.	CMJ height (cm) 39.5 ± 6.7 → 36.9 ± 5.3 (*P*=NA, *d*=0.43), -6.5% 1RM-SQ (kg) 172 ± 30 → 152 ± 21 (*P*=NA, *d*=0.77), -11.5% *Analysis of descriptive data (with percent change) only.*	Individual variation in the results exist; broadly, ↓ overall training workloads (-64%); lower HRV, and ↓ physical fitness.

1RM – one-repetition maximum; BCA – Body Composition Analysis; BFR – blood blow restriction; BIA – bioelectrical impedance analysis; BIVA – Bioelectrical Impedance Vector Analysis; BMI – body mass index; CMJ – countermovement jump; CON PT – concentric peak torque; CT – continuous training; D – dominant; ECC PT – eccentric peak torque; ER – external rotation; FB – Flamingo balance; HGS – Handgrip strength; HIIT – high intensity interval training; HQ Ratios – Hamstrings to Quadriceps Ratio; HR – heart rate; HRV – Heart Rate Variability; I.ae./I.an. Threshold – individual aerobic/anaerobic threshold; IET – increment exertion test; IFT – intermittent fitness test; IR – internal rotation; KE – knee extensor; KF – knee flexor; LIT – low-intensity interval training; LSI – limb symmetry index; MBT – medicine ball throw; MIIT – moderate-intensity interval training; MPV – mean propulsive velocity; MR – Mean Rank; NA – not applicable; ND – non-dominant; PD – performance difference; PhA – phase angle; Rel. Strength – relative strength; RFD – rate of force development; R/H – resistance adjusted for stature; ROM – range of motion; RSImod – reactive strength index modified; RT – resistance training; SJ – squat jump; SLJ – standing long jump; SQ – squat; VMA – (velocità massima aerobica) maximum aerobic speed; Xc/H – reactance adjusted for stature; Yoyo-IRT1 – YoYo Intermittent Recovery Test.

Note: Selected results of performance (e.g., jump, sprint, throw) and body composition parameters are shown; with additions of significant results in non-performance variables (e.g., ER/IR ratio); **P* values in bold are statistically significant.

TABLE S2. Summary of findings according to the GRADE framework for the quality of evidence.

Outcomes	Certainty assessment								Effect	Certainty	Importance
	No. of studies (n ESs)	Study design	Risk of bias	Inconsistency	Indirectness	Imprecision	No. of participants	Publication bias	Absolute (95% CI)		
Body mass	10 (11)	Obs.[a]	Not serious[b]	Not serious[c]	Not serious	Very serious[f]	208[g]	Very serious	SMD= −0.115 95% CI= −0.214 to 0.164[f]	⊕◯◯◯ Very low	CRITICAL
CMJ height	10 (10)	Obs.[a]	Not serious[b]	Serious[d]	Not serious	Very serious[f]	159[g]	Not serious	SMD= −0.308 95% CI= −0.671 to 0.056	⊕◯◯◯ Very low	CRITICAL
CMJ peak power	6(6)	Obs.[a]	Not serious[b]	Not serious[e]	Not serious	Serious[g]	87[g]	NA[h]	SMD = −0.197 95% CI= −0.362 to −0.032[f]	⊕◯◯◯ Very low	CRITICAL

CI: confidence interval; **Obs.**: observational; **SMD:** standardized mean difference; **ES**: effect size; **NA**: not applicable. **Explanations** a. Low level of evidence because of the observational nature of the included studies.; b. All included studies had a low risk of bias.; c. Eight out 10 ESs reported no significant change in body mass due to the COVID-19 lockdown; Statistical heterogeneity was significant, with country partially accounting for the observed variation.; d. Six ESs indicated no significant effect of the COVID-19 lockdown on CMJ height, while three showed a significant decrease, and one reported a significant increase; The results were heterogeneous, with an I^2 value exceeding 75%, partially attributed to differences in sample size.; e. All ESs showed no significant effect of COVID-19 lockdown on CMJ peak power, with no significant heterogeneity; f: Two downgrades were performed because the total number of participants < 400 and the wide CI (crosses the zero).; g. A downgrade was performed because the total number of participants < 400; h: It is not possible to assess publication bias due to the limited number of included studies.

Articles published in the Biology of Sport are licensed under an open access Creative Commons CC BY 4.0 license.
